# Working Memory Predicts Hypothalamus-Pituitary-Adrenal Axis Response to Psychosocial Stress in Males

**DOI:** 10.3389/fpsyt.2020.00142

**Published:** 2020-02-28

**Authors:** Li Lin, Jianhui Wu, Yiran Yuan, Xianghong Sun, Liang Zhang

**Affiliations:** ^1^Key Laboratory of Behavioral Science, Institute of Psychology, Chinese Academy of Sciences, Beijing, China; ^2^Department of Psychology, University of Chinese Academy of Sciences, Beijing, China; ^3^Center for Brain Disorder and Cognitive Science, Shenzhen University, Shenzhen, China; ^4^Shenzhen Institute of Neuroscience, Shenzhen, China

**Keywords:** cortisol, acute stress, working memory, ERP, P2

## Abstract

The hypothalamic-pituitary-adrenocortical (HPA) function is crucial for adaptation to stress and recovery of homeostasis. Physiological alteration in the HPA axis has been shown to play a pivotal role in the generation of stress-related disorders. A growing number of studies have begun to identify which variables are possible to predict individual HPA response and associated stress vulnerability. The current study investigated the relationship between working memory and the subsequent magnitude of HPA response to psychosocial stress in a non-clinical population. Working memory was assessed utilizing an n-back task (2/3-back) in thirty-nine healthy young men, whose electroencephalograms were recorded. The HPA response was measured using the percentage increase in cortisol to an acute psychosocial stress protocol called the Trier Social Stress Test (TSST). Our results show that longer reaction time and smaller amplitude of P2 predict a relatively lower HPA response to stress. Our study provides new insights into how neurocognitive factors can be used to predict HPA response to acute stress.

## Introduction

Stress is familiar to people in modern life and chronic exposure to stress may lead to mental and physical disorders. To respond to stressful challenges, the brain has developed crucial neural and neuroendocrine mechanisms (1). Among these mechanisms, a stress response system based on the hypothalamic-pituitary-adrenocortical axis (HPA axis) is one important pathway that regulates stress hormone levels (2). The activation of the HPA axis results in an increase in circulating glucocorticoids levels, which in humans primarily comprises cortisol (3). From the physiological perspective, the HPA response triggered by acute stress is crucial for adaptation to stress and recovery of homeostasis, up-regulating the hormone cortisol to cope with challenges when at risk but down-regulating cortisol through a negative feedback loop when challenges have been resolved (4). Since the HPA axis is the key system to mobilize the organism's resources to deal with threat through regulating cortisol levels (5), it provides a critical mechanism for survival.

Chronic activation of HPA axis exerts adverse effect on the development of brain structures and neuroendocrine systems. Physiological alteration in the HPA axis has been shown to play a pivotal role in the generation of stress-related disorders, including but not limited to depression, anxiety and cardiovascular diseases (6). The focused review by Handwerger (7) has described the patterns of HPA-axis reactivity under stress in healthy individuals compared to those with stress-related disorders, i.e., post-traumatic stress disorder (PTSD) and major depressive disorder (MDD). In PTSD studies, some researchers have found that stress can trigger greater cortisol reactivity in anticipation of a stressor in PTSD patients (8, 9). In MDD studies, researchers have found that the HPA responses to acute stress in MDD was similar to controls (10) or relatively blunted (11, 12). In the recent decade, blunted cortisol response was found in adolescents with moderate or severe depression (13) and soldiers who showed greater increase in PTSD symptomatology (14). Although it remains difficult to draw a clear conclusion about the relationship between HPA response and stress-related disorders, the majority of studies has supported the viewpoint that the phenomenon of hypocortisolism, a relatively decreased cortisol reactivity, is related to pathophysiology of stress-related disorders (15, 16).

Individuals differ markedly in their vulnerability to stressful challenges (17), but little is known about what kind of individuals are at higher risk for the generation of HPA dysregulation and associated stress-related pathologies. The past decade has observed a growing number of studies using demographic variables (18, 19), personality traits (20, 21), and exposure to adversity (22) to predict physiological responses to laboratory psychosocial stress [for reviews see (6, 23)]. Recent studies have shown that the better performance of cognitive functions under non-stressful situation can also predict stronger HPA response to acute stress. For example, poor cognitive abilities, including episodic memory and reasoning ability, have been observed to be associated with a blunted HPA response to acute stress (24, 25). In addition, attentional bias toward negative and social stress stimuli was also suggested to present a useful tool to predict an increased cortisol response to acute stress (26, 27). Furthermore, two studies found that the neural activity of error consciousness—error-related negativity (ENR) and error positivity (Pe)—can predict stress reactivity by measuring Event-related potentials (ERPs) (28, 29).

Although previous studies have shown that the behavioral measures of cognitive control predict HPA response or cortisol reactivity to acute stress [e.g., (24, 25, 30)], there are relatively fewer neurocognitive indicators. To our knowledge, only two ERP studies have obtained preliminary outcomes in ERN and Pe potentials (28, 29); therefore, further evidence in other cognitive components is still needed. Especially, the executive function has been reported to be closely related to stress response [for a review (31)]. However, it remains unknown whether the neurocognitive activity of executive function can serve as predictors of HPA response. Working memory represents a core executive function, both holding and actively operating with information through directing attention to task-related activity. Extensive research has shown that working memory is closely related to the function of prefrontal cortex (PFC) (32), which is also one of the brain areas regulating the magnitude of HPA response to stress (33). Thus, the current study was aimed at investigating the possibility of working memory to serve as a predictive indicator of HPA response to acute stress. More importantly, we were interested in whether and how the executive component of working memory and its related ERPs predicts HPA response.

N-back task is a classic paradigm to measure the executive component of working memory in neurocognitive studies (34). For this task, two typical ERP components P2 and P3 have been frequently analyzed to assess the attentional process and maintenance process in working memory (35). The P2 component is most prominent at frontal-central sites and is believed to be associated with early attention allocation and the initial onset of context updating (36, 37). In addition, the P3 component is a centro-parietal positive component that reflects cognitive resources or capacity of working memory (38, 39).

The current study used the N-back task to assess working memory under non-stressful situation, and aimed at examining whether and how the behavioral performance and ERP correlates of working memory is related to the HPA response induced by an acute psychosocial stress protocol. We hypothesized that better working memory performance positively predicts HPA responses. Furthermore, we tested whether the ERPs (P2 and P3 components) are associated with the HPA response to stress.

## Materials and Methods

### Participants

Forty healthy Chinese male college students aged 18–27 years old were recruited from universities in Beijing in the present study. We only recruited male participants due to the potential influences of the menstrual cycle and oral contraceptives on cortisol levels (40). To control for potential influences on stress hormones, we strictly followed the general exclusion criteria in acute stress studies: (1) history of chronic physiological or endocrine disease; (2) history of psychiatry or neurological disorder; (3) use of medication within 2 weeks before the experiment; (4) chronic overnight work or circadian rhythm disorder; (5) excessive alcohol consumption (more than two alcoholic drinks per day) or tobacco use (more than five cigarettes per day); and (6) current periodontitis. Due to extremely high cortisol response (over 2.5 SD), one participant was excluded, resulted in 39 participants. Their average age was 21.92 (*SD* = 2.08) years old with three missing values, and their average education level was 15.46 (*SD* = 1.52) years with two missing values. All participants provided informed consent and were offered monetary reimbursement for their participation. This study was approved by the Ethics Committee of Human Experimentation in the Institute of Psychology, Chinese Academy of Sciences.

### Procedure

**Figure 1:**
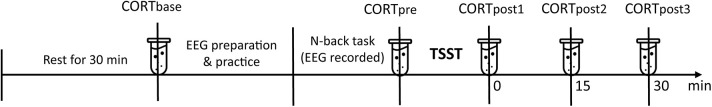
Timeline of the procedure. Salivary cortisol (CORT) samples were collected at the baseline, before the Trier Social Stress Test (TSST), 0/15/30 min after the TSST.

The timeline of our procedure is shown in Figure 1. Experiments were conducted in the afternoon to control for the circadian rhythm of cortisol levels (41, 42). Participants were instructed to refrain from smoking, eating, drinking anything but water, and doing exercise 2 h before the study. The participants arrived at the laboratory in the afternoon either at 1:30 or 3:30 p.m. Upon arrival, participants were required to rest in a quiet room for 30 min while filling out a questionnaire (see section Questionnaire). Salivary samples (CORT) were collected after the rest phase. Following preparation of electroencephalograms (EEG), the participants practiced on the n-back task (see section N-Back Task) until they reached 80% accuracy (43). Salivary sample was collected again before the TSST task to check whether the n-back task had made the participants stressed. The participants then completed the TSST task (see section Stress Induction). Salivary samples were collected at 0, 15, and 30 min after the end of the TSST task.

### Questionnaire

#### Neuroticism

As neuroticism has been proved to be closely related to physiological stress response (44, 45), we included it as a control variable in our research. Participants completed an 8-item neuroticism scale from the Big Five Inventory (46). Participants responded on a Likert 5-point scale (ranging from 1 = strongly disagree to 5 = strongly agree). The scale has been widely used and validated. In this study, Cronbach's alpha coefficient for neuroticism was 0.77.

### N-Back Task

A numerical n-back task (*n* = 2, 3) was used to assess the working memory. White one-digit numbers (from 1 to 9) were presented on a black background screen 60 cm away from the participants' eyes, at a visual angle of (1 × 2). Each number was displayed for 500 ms with a randomly varied inter-stimulus interval of 1,300–1,700 ms. The practice blocks consisted of 20 trails for each load. The experimental blocks consisted of 100 trials for each load, with 50% match-trials. The test lasted approximately 10 min in total, including instruction, training and experimental blocks. Participants had to indicate whether the number appeared on the screen matched the one presented n-trials back, and to respond by pressing the “match”/“non-match” button with their right or left index finger as quickly as possible. The “match”/“non-match” button was counterbalanced for the left/right hand.

### EEG Recordings

Electroencephalograms (EEG) were recorded from 64 sites according to the international 10–20 system (Neuroscan Inc., USA), on-line referenced to the left mastoid. The vertical electrooculogram (VEOG) was recorded by two electrodes located above and below the left eye. The horizontal electrooculogram (HEOG) was recorded using two electrodes at about 1 cm from the outer canthus of each eye. The impedance was kept below 5 kΩ. The EEG signals were amplified with band-pass filter (0.05–100 Hz), with a sampling rate of 1,000 Hz.

The EEG data was processed off-line using Scan 4.3 (Neuroscan Inc., USA). The data was re-referenced to the average of the left and right mastoids. Ocular artifacts were identified and corrected using a regression algorithm implemented in the Neuroscan software (47). The data was digitally lowpass-filtered with 30 Hz, before being segmented into epochs of 1,200 ms, including a 200 ms pre-stimulus baseline, and time-locked to the onset of each stimulus. Trials with artifacts exceeding ±100 μV were rejected.

### Stress Induction

The TSST task in the current study was a modified version (48, 49) of the original TSST task (50), including a 5-min preparation, a 5-min speech, and a 5-min mental arithmetic task. In the preparation stage, participants were told that they were accused of shoplifting and had to prepare a defense in front of the store managers and a police officer. The participants were then escorted to a room where three experimenters (two females and one male) in white coats were waiting. The experimenters kept a neutral facial expression through the speech and mental arithmetic task. The participants stood in front of the experimenters and spoke to a microphone. After the speech, the participants had to subtract 13 from 1,022 orally and were required to start from 1,022 again if they made a mistake. The speech and arithmetic task stages were videotaped.

### Physiological Measures

The salivary samples were collected using Salivette collection tubes (Sarstedt, Rommelsdorf, Germany). Salivary samples were frozen at −22°C within 2 h of collection until analysis. For analysis, samples were thawed and centrifuged at 3,500 rpm for 15 min and then analyzed using an electrochemiluminescence immunoassay (ECLIA, Cobas e 601, Roche Diagnostics). The lower sensitivity for cortisol was 0.500 nmol/L. Intra- and inter-assay variations were below 10%.

### Data Analysis

Behavioral performance was evaluated by measuring by accuracy (ACC) and reaction time (RT). Incorrect responses or RTs below 100 ms were excluded from both behavioral and ERP analyses. For ERP analysis, both matched and non-matched trials were included in the averaging of P2 and P3 components (43, 51). In our study, Fz and Pz were the sites that showed the largest amplitudes of P2 and P3, respectively. The mean amplitude of P2 was calculated 160–190 ms after the onset of stimuli. The mean amplitude of P3 was calculated 300–450 ms after the onset of stimuli.

In order to check whether stress induction was successful, one-way repeated measures analysis of variance (ANOVA) was conducted for salivary cortisol, with time as a within-subject measure. Greenhouse-Geisser corrected *p*-values were reported when sphericity assumptions were violated. *Post-hoc* comparisons were conducted using Bonferroni adjustments. Partial η^2^ was provided as a measure of the effect size.

The magnitude of HPA response to acute stress was indexed by the percentage change in salivary cortisol (18, 52), which was calculated as follows: percentage cortisol increase = (CORT_peak_ – CORT_base_)/CORT_base_. The peak point was defined according to the group maximum point among the three post-TSST measures. The “percentage cortisol increase” was phrased as “cortisol reactivity” in the following to make the expression more concise.

Bivariate correlations were conducted among all potential predictor variables (behavioral and ERP index), outcome variable (cortisol reactivity), and controlled variables (age and neuroticism). For those behavioral and ERP index that were significantly correlated with cortisol reactivity, they were further included in a hierarchical regression in step 2 to examine how working memory predicts HPA response, with age and neuroticism being entered in Block 1 as control variables.

## Results

### Descriptive Data

**Figure 2:**
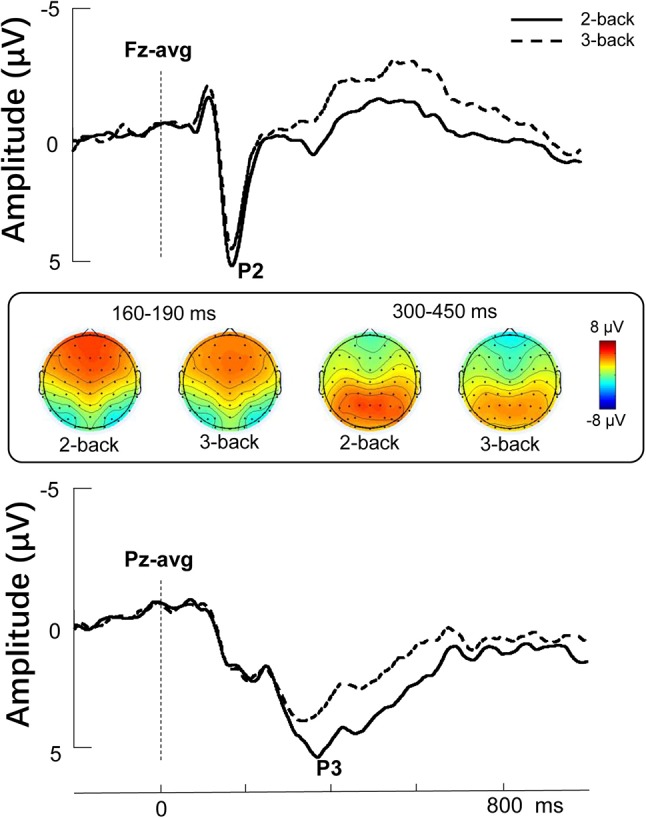
Stimulus-locked grand average ERP amplitude (μV) elicited during the 2-back and 3-back conditions. Topographies represent the scalp distributions of the P2 (160–190 ms) and P3 components (300–450 ms).

The descriptive behavioral and ERP data of N-back task were presented in Table 1. To evaluate the difference between 2- and 3-back, we performed a paired *t*-test. Our analysis showed that the accuracy of 2-back was significantly higher than that of 3-back (*t* = 12.546, *p* < 0.001), and the reaction time of 2-back was also significantly shorter than that of 3-back (*t* = −2.503, *p* = 0.017). The Grand-average ERPs are shown in Table 1 and Figure 2. The difference in P2 amplitudes between 2- and 3-back were not significant (*t* = 1.895, *p* = 0.066). The P3 amplitudes were significantly larger in 2-back than 3-back (*t* = 3.286, *p* = 0.002).

**Table 1:** Descriptive statistics of study variables.

**Variable**	**Mean**	**Standard deviation**
Age	2.72	0.60
Neuroticism	22.07	2.14
Behavioral measures		
2-back ACC	0.92	0.05
2-back RT	775	158
3-back ACC	0.78	0.08
3-back RT	860	162
ERP measures		
2-back P2	4.79	4.23
3-back P2	4.36	4.03
2-back P3	3.94	4.00
3-back P3	3.25	3.09
Percentage cortisol increase	0.88	1.18

**Figure 3:**
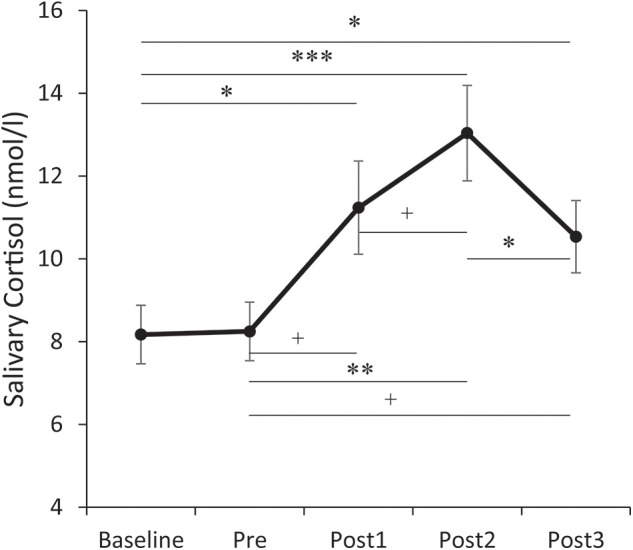
Temporal changes of salivary cortisol levels. Error bars represent standard errors of the mean. Baseline: after 30 min rest phase. Pre: immediately before the Trier Social Stress Test (TSST). Post 1/2/3: at 0/15/30 min after the TSST. ^†^*p* < 0.1, *p < 0.05, **p < 0.01, and ***p < 0.001.

For salivary cortisol (see Figure 3), the repeated measures ANOVA showed a significant main effect for Time, *F*_(4, 152)_ = 12.053, *p* < 0.001, partial η^2^ = 0.241. *Post-hoc* analysis showed that the salivary cortisol levels collected at 0 min, 15 min, and 30 min post-TSST were significantly higher than baseline (*ps* < 0.05). The salivary cortisol level collected immediately before the TSST was not significantly different from the baseline (*p* > 0.1). The salivary cortisol level collected 0 min post-TSST was not significantly different from either 15 min or 30 min post-TSST (*ps* > 0.05). The salivary cortisol level collected 15 min post-TSST was significantly higher than 30 min post-TSST (*p* = 0.013). As 15 min post-TSST showed the group maximum value among the three post-TSST measures, the cortisol reactivity was calculated as follows: percentage cortisol increase = (CORT_15min_ – CORT_base_)/CORT_base_.

### Bivariate Correlation

Next, we performed a bivariate correlation analysis to identify the variables for subsequent regression analysis (Table 2).

**Table 2:** Bivariate correlations among study variables.

**Variable**	**1**	**2**	**3**	**4**	**5**	**6**	**7**	**8**	**9**	**10**
1. Age	—									
2. Neuroticism	−0.147	—								
3.2-back ACC	−0.115	−0.129	—							
4.2-back RT	0.081	−0.066	−0.056	—						
5.3-back ACC	−0.129	−0.185	0.672^**^	0.018	—					
6.3-back RT	−0.042	0.193	0.054	0.472^**^	−0.047	—				
7.2-back P2	−0.031	0.260^*^	0.093	−0.253	0.110	−0.209	—			
8.3-back P2	−0.159	0.157	0.274^*^	−0.181	0.242	−0.176	0.849^**^	—		
9.2-back P3	0.133	−0.271^*^	0.027	−0.256	0.084	0.027	0.159	0.143	—	
10.3-back P3	0.128	−0.374^**^	0.126	−0.045	0.324^*^	−0.144	0.004	0.084	0.703^**^	—
11. Percentage cortisol increase	0.211	0.072	0.061	−0.372^*^	−0.057	−0.092	0.296^†^	0.365^*^	0.166	0.157

† *p < 0.1*,

* p < 0.05, and

** *p < 0.01*.

**Figure 4:**
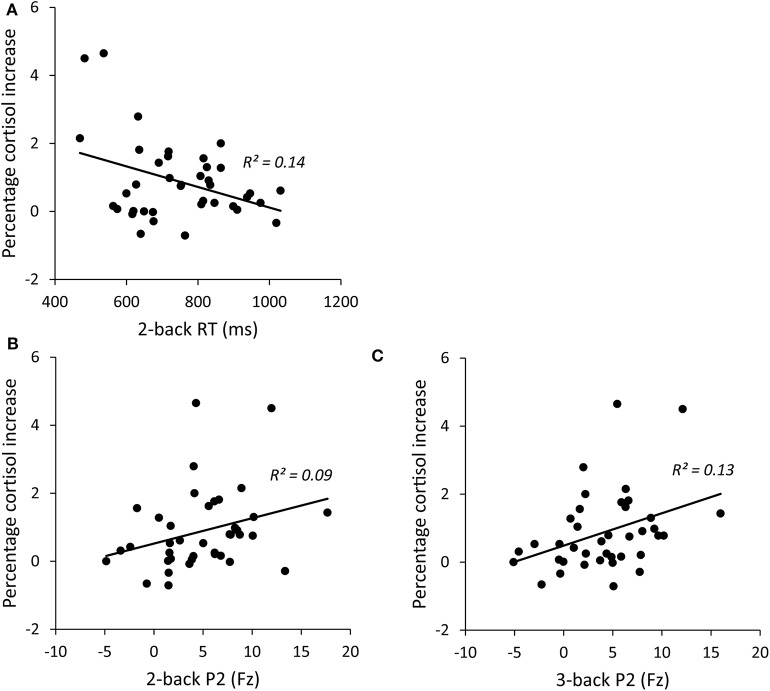
Correlations between percentage cortisol increase and 2-back RT **(A)**, 2-backP2 amplitudes **(B)**, and 3-backP2 amplitudes **(C)**. RT, reaction time.

As shown in Figure 4, the reaction time of 2-back was significantly correlated with the cortisol reactivity, *r* = −0.372, *p* = 0.020. Among the ERP indices, the P2 amplitudes of 2-back were marginally associated with the cortisol reactivity, *r* = 0.296, *p* = 0.068, and the P2 amplitudes of 3-back were significantly associated with the cortisol reactivity, *r* = 0.365, *p* = 0.022 (see Figure 4). The accuracy and P3 amplitudes under both 2-back and 3-back as well as the reaction time of 3-back were not significantly related to cortisol reactivity (*ps* > 0.1).

### Regression Analysis

In order to examine how working memory predicts HPA response, we performed hierarchical regression analysis using the cortisol reactivity as the dependent variable and the three working memory indices that are significantly associated with cortisol reactivity as predictors. Age and neuroticism were firstly entered as controlled variables in the block 1. 2-back index (2-back RT and P2 amplitude under 2-back) was entered in the block 2 and 3-back index (P2 amplitude under 3-back) was entered in the block 3. Table 3 presents hierarchical regression analysis results for the prediction model. After controlling for the impact of age and neuroticism (Block 1), measures of 2-back predicted marginally significant additional variance (Δ*R*^2^ = 0.191). Specifically, reaction time of 2-back in Block 2 significantly predicted cortisol reactivity (β = −0.379, *t* = −2.180, *p* = 0.037). Next, after controlling for 2-back measures, 3-back index accounted for significant additional variance in the prediction model (Δ*R*^2^ = 0.139). P2 amplitudes of 3-back significantly predicted cortisol reactivity (β = 0.803, *t* = 2.546, *p* = 0.016). Note that the enter of 3-back P2 in block 3 led to suppressor effect on 2-back P2 due to the inevitable high correlation between 2-back P3 and 3-back P2 (*r* = 0.849). However, 2-back P2 was a positive predictor to cortisol reactivity in the similar pattern as 3-back P2, as shown in Figure 4.

**Table 3:** Regression analysis of WM index predicting HPA response.

	**Percentage cortisol increase**						
	**Predictor**	***B***	***t***	***B***	***t***	***B***	***t***
Block 1	Age	0.113	1.129	0.092	0.939	0.131	1.436
	Neuroticism	0.186	0.587	0.342	1.135	0.626	2.096^*^
Block 2	2-back RT	—	—	−0.003	−2.180^*^	−0.003	−2.753^*^
	2-back P2	—	—	0.037	0.821	−0.148	−1.764^†^
Block 3	3-back P2	—	—	—	—	0.21	2.546^*^
Δ *R^2^*		0.045	0.191^†^	0.139^*^			
Model *F*		*F*_(2, 32)_ = 0.746	*F*_(4, 30)_ = 2.313^†^	*F*_(5, 29)_ = 3.485^*^			

† p < 0.1 and

* *p < 0.05*.

*The Cook's distances of the two highest cases in the regression are 0.467 and 0.221, respectively*.

## Discussion

The current study attempted to examine whether and how working memory (indexed by both behavioral performance and ERP correlates) predicts HPA response to the acute psychosocial stress. Our results show that the longer reaction time of 2-back and the smaller P2 amplitudes elicited during both 2-back and 3-back task predicted blunted HPA response.

The behavioral outcome showed that a longer reaction time at 2-back predicts smaller cortisol reactivity, indicating that individuals who respond slower to stimuli also tend to exhibit relatively blunted HPA reactivity when encountering stressful events. Our behavioral finding is in agreement with previous studies that suggested that cognitive abilities are positively correlated with cortisol reactivity to stress (24, 25). For example, Ginty et al. (24) found that fora middle-aged population, the performance in the episodic memory task (immediate and delayed recall) positively predicts the cortisol reactivity to acute stressors. In agreement with this finding, Slattery et al. (25) found that among the assessments of IQ, academic achievement and memory, only poorer memory performance (assessed with a Story Memory subtest) predicted lower cortisol reactivity to psychosocial stress. These findings strongly suggest that better performance of cognitive task predicts stronger cortisol reactivity. Stawski et al. (53) also found that higher cognitive function, particularly executive function, was associated with healthier daily cortisol profiles as reflected in steeper diurnal cortisol slope, higher morning cortisol levels, and lower afternoon and evening cortisol levels. Our study provides further evidence to support that the fundamental cognitive abilities predict the function of HPA system with a more objective indicator. The possible explanation for this correlation might be that individuals who are faster-reactors, or more generally, who with better executive functions, are more capable to efficiently mobilize their cognitive resources to cope with the stress and demands of daily life (18, 25) so that their HPA function are less influenced and tends to be retained in a better condition. Accordingly, individuals who are weak in neurocognitive abilities may experience more stress in their daily life (25, 54, 55), resulting in a chronic ongoing activation of the HPA axis.

However, we did not found correlation between other three behavioral measures and HPA reactivity to acute stress except the reaction time of 2-back. We assume that it could be the speed rather than the accuracy of the behavioral response that is associated with stress reactivity. In accordance with this assumption, a couple of previous studies investigating the effect of stress on working memory also reported that stress affected the reaction time instead of the accuracy in N-back task and Sternberg working memory test (56, 57). However, the absence in correlation between 3-back RT and cortisol reactivity might derive from its challenging load. The above results may also suggest that even for the same type of cognitive task, not every measure can predict the reactivity to stress.

Moreover, another finding of our study is that the amplitudes of the P2 component positively predicts the magnitude of HPA response. It is generally accepted that P2 reflects early attention allocation and stimulus detection (37). Previous ERP studies on working memory suggested that P2 reflects the early stages involved in information selection (58, 59) and the initial onset of context updating (32). The regression analysis performed in our study indicates that the efficiency of allocating attention to stimuli and selecting relevant information in the early stage of WM processing is associated with HPA response to acute stress. In contrast, we found that the P3 amplitude failed to predict stress-related HPA responses. One possible interpretation is that while P2 is most prominent in the frontal-central sites (60), P3 is mainly central-parietal. As the PFC is thought to be the main brain area that regulates the magnitude of HPA response to stress (33), the P2 component might tend to be a more sensitive predictor. The other explanation lies in the functional difference between P2 and P3 components. The P2 component is thought to be associated with early attention allocation and stimulus detection (37), while the P3 component generally reflects cognitive resources or capacity of working memory (38, 39). The likely reason is that the effective predictor of HPA response is related to the neural activity of early attention process of working memory rather than the later processing stages.

Our findings suggest that the mechanism underlying working memory and HPA response shares an overlapping neural circuit. While previous studies on the effect of acute stress suggested that working memory is sensitive to the stress-induced cortisol increase (61–63), our study clearly showed that working memory (by way of behavioral performance and ERP correlates) is a suitable predictor of HPA response to acute stress. Taken together, these findings provide accumulated evidence that the cognitive function is not only affected by the stress-induced cortisol, but also can be used to predict cortisol reactivity to stress.

Our study has a number of limitations to be addressed. First, the psychosocial stress applied to the participants in the laboratory is moderate and temporary, and thus the results of our study might not be compared with the more stressful events under real-life conditions. Second, since our study sample is based on young males, the findings cannot be generalized to females and other age groups in absence of further evidence.

This study provided several clinical and research implications. First, our findings support that working memory has the potential to serve as a neurocognitive marker of the HPA responses to acute stress, providing new avenues to identify stress vulnerability. Second, the findings enhance our understanding of the relationship between cognitive neural mechanisms of working memory and stress-related HPA response system. Finally, the findings may help us with the early identification of stress-related disorders so that they can be more effectively prevented or intervened. Improved training of working memory might enhance neurocognitive abilities and further prevent disruptive alterations in the functioning of the HPA axis, thus preventing mental complications for patients affected.

## Data Availability Statement

The datasets generated for this study are available on request to the corresponding author.

## Ethics Statement

The studies involving human participants were reviewed and approved by Ethics Committee of Human Experimentation in the Institute of Psychology, Chinese Academy of Sciences. The patients/participants provided their written informed consent to participate in this study.

## Author Contributions

LL contributed to study design, data collection, data analysis, and wrote the manuscript. JW participated in the revision of the manuscript. YY participated in the design of the n-back paradigm. XS participated in the revision of the manuscript. LZ supervised the whole study and was responsible for the study design, data interpretation, and the revision of the manuscript. All the authors approved the final manuscript.

### Conflict of Interest

The authors declare that the research was conducted in the absence of any commercial or financial relationships that could be construed as a potential conflict of interest.
